# Investigating Intracerebral Hemorrhage Evacuation Procedure Volumes Before and After the Minimally Invasive Surgery Plus Alteplase for Intracerebral Hemorrhage Evacuation III (MISTIE III) Trial

**DOI:** 10.7759/cureus.112914

**Published:** 2026-07-18

**Authors:** Graham A Branscom, Evan M Ren, Keshv Krishna Srinivasan, Michael B Lemonick, Matthew T Carr, Leilei Hao, Akhil Rao, Tanvir F Choudhri, Christopher P Kellner, John Y.K. Lee

**Affiliations:** 1 Neurosurgery, Icahn School of Medicine at Mount Sinai, New York, USA; 2 Neurosurgery, University of Pennsylvania Perelman School of Medicine, Philadelphia, USA

**Keywords:** hemorrhagic stroke, intracerebral hemorrhage, medical insurance, minimally invasive surgery, randomized controlled trial

## Abstract

Introduction

Intracerebral hemorrhage (ICH) constitutes 10-15% of all strokes in the United States and has a 30-40% mortality rate. The Minimally Invasive Surgery Plus Alteplase for Intracerebral Hemorrhage Evacuation III (MISTIE III) trial showed no significant functional benefit of minimally invasive catheter drainage (MICD) and postoperative thrombolysis compared to medical management. Our objective is to evaluate how the results of the MISTIE III trial corresponded to changes in ICH evacuation procedure volumes.

Materials and methods

We used an aggregated dataset containing Medicare Part B procedure data from 2013 to 2023. We calculated the normalized volume of MICD procedures, supratentorial craniotomies, and infratentorial craniotomies for ICH evacuation. We normalized the volumes by calculating the percentage of all neurosurgical procedures that included ICH evacuation for each year. We used an interrupted time series (ITS) to find statistically significant changes in volumes before (β_pre_) and after (β_post_) 2019, when the MISTIE III results were published.

Results

Normalized MICD volumes increased slightly before 2019 (β_pre_ = 0.0001, p* *= 0.022). After 2019, MICD volumes increased more dramatically over the long term than would have been expected based on the pre-2019 trend (β_post_ = 0.0028, p < 0.001). However, supratentorial and infratentorial craniotomy volumes, as well as overall ICH evacuation volumes across all procedure types, showed no significant long-term change (p = 0.091, p = 0.18, p = 0.68, respectively).

Conclusions

The results of MISTIE III did not show a benefit of MICD over medical management for ICH with respect to the primary outcome of functional independence. We found that, despite these results, MICD evacuation procedure volumes increased significantly after the publication of the MISTIE III results. However, this association is only correlative, and other factors besides MISTIE III could have affected procedure volumes.

## Introduction

Spontaneous intracerebral hemorrhage (ICH) is a life-threatening condition that constitutes around 10-15% of all strokes in the United States [1]. ICH most commonly occurs in deep brain structures when the small vessels supplying these areas are damaged and rupture. Risk factors include hypertension, cerebral amyloid angiopathy, coagulopathies, thrombocytopathies, and anticoagulant therapy [1,2]. Over the past two decades, the observed incidence of ICH has doubled to affect more than 80,000 Americans annually, and despite advances in treatment, the condition carries a 30-40% mortality rate [1]. Patients who survive the initial episode are at risk for recurrent stroke and severe complications, most commonly due to hematoma formation and cerebral edema [1]. As such, rapid and proper management of this condition is paramount to preventing further neurological decline and death [1].

The main goal of acute treatment is to limit hematoma expansion. Meeting this aim requires multiple, concurrent treatments in a “bundle of care” approach, which has been shown to maximize patient recovery [3]. First-line medical management includes rapid reversal of anticoagulation, blood pressure control, and stroke unit protocols, such as glucose control and antipyretics [4]. In addition to medical therapy, select patients undergo hematoma evacuation, although evidence supporting this strategy has been mixed over time [1,3]. The Surgical Trial in Intracerebral Hemorrhage (STICH) series of clinical trials found that surgical intervention, often via an open craniotomy, provided no significant benefit over medical management alone [5,6]. STICH II showed a small survival benefit for patients with a spontaneous ICH without intraventricular hemorrhage [6].

In response to the negative results from the STICH trials for open craniotomy, the Minimally Invasive Surgery Plus Alteplase for Intracerebral Hemorrhage Evacuation II (MISTIE II) trial was designed as a minimally invasive surgery (MIS) approach to ICH evacuation. MISTIE II established a safety profile for MIS similar to that of craniotomy but did not show a significant difference in the primary outcome [7]. Then, the MISTIE III trial examined stereotactic catheter placement into the hematoma and postoperative intraclot thrombolysis with alteplase compared with standard medical management alone [8]. Their study found no significant functional benefits; however, the treatment group had lower mortality and adverse events at one year [8]. As such, MIS is safer than open craniotomy despite lacking an obvious functional advantage, as reflected in the American Heart Association (AHA) and American Stroke Association (ASA) treatment guidelines [9].

Given the prevalence of ICH and its potentially serious complications [1], along with evolving treatment guidelines regarding the use of MIS for ICH evacuation [9], there is a need to characterize contemporary procedure volumes. Additionally, analyzing the relationship between randomized controlled trials (RCTs) and procedure volumes is critical to understanding how academic research affects clinical practice. This study seeks to investigate the temporal trends in the volumes of MIS and craniotomies for ICH treatment among Medicare patients following the publication of MISTIE III. We hypothesize that MIS ICH evacuation volumes would decrease following the publication of MISTIE III.

## Materials and methods

Identification of trials

We searched the neurosurgical literature for all RCTs investigating ICH evacuation between 2014 and 2022. As our dataset of procedure volumes includes surgeries from 2013 to 2023, this time frame for evaluating RCTs investigating ICH evacuation allows for a one-year follow-up. Our inclusion criteria included any RCT comparing ICH evacuation (craniotomy or MIS) versus medical management. Our exclusion criteria included any study published outside of 2014 to 2022. This allowed us to statistically identify how trends in procedure volumes changed before and after the RCT publication date. Only one such trial, MISTIE III, which was published in 2019 [8], met these criteria.

Data source

We analyzed the “Medicare Physician and Other Practitioners by Geography and Service” dataset, which is publicly available from the Centers for Medicare and Medicaid Services [10]. The dataset reports volume data from Original Medicare Part B physicians. The data includes the year, state, current procedural terminology (CPT) code, and number of services. The data includes procedure volumes from 2013 to 2023 (inclusive).

Data filtering and analysis

We filtered the dataset to include only CPT codes for ICH evacuation procedures. These included craniotomy approaches (61313 for supratentorial and 61315 for infratentorial) and burr hole(s) with minimally invasive catheter drainage (MICD) (61156). We identified these CPT codes by examining their descriptions in the American Academy of Professional Coders (AAPC) [11]. We then calculated the raw number of each of these procedures that were billed per year.

In addition to the raw procedure volumes, we also found normalized procedure volumes. We calculated these by filtering the dataset for all the primary CPT codes used in neurosurgical procedures. This included 406 primary CPT codes in total and notably excluded add-on CPT codes such as 61781 (stereotactic navigational planning) and 69990 (operative microscope for microdissection), since these codes are added to primary procedure CPT codes to indicate extra services. For each year, we divided the number of ICH evacuation procedures by the total number of primary CPT codes billed for all neurosurgical procedures.

Statistical tests

We used an interrupted time series (ITS) to compare normalized ICH evacuation procedure volumes before and after the publication of the MISTIE III trial in 2019. ITS is a form of segmented regression that allows identification of the directionality and significance of changes in the normalized procedure volumes before and after 2019. In the ITS model, the percentage of total services is modeled using an ordinary least squares segmented regression equation that includes the linear coefficients β_pre_, β_post1_, and β_post2_. β_pre_ models the percentage of total services before the RCT year. β_post1_ compares the observed post-RCT year level to the counterfactual level predicted by the pre-RCT year trend. In other words, β_post1_ tests whether the normalized procedure volume after the RCT publication year was higher or lower than expected in that year, assuming the trend from before the RCT continued. β_post2_ is similar to β_post1_, but it measures long-term changes after the RCT year by comparing the post-intervention line slope to the counterfactual slope (β_pre_). Therefore, β_pre_ models the trend before the RCT, β_post1_ models the value in the year after the RCT, and β_post2_ models the long-term change after the RCT. Each of these coefficients has an associated p-value that was calculated using the ordinary least squares t-test. We used a p-value cutoff of 0.05.

Software

All analyses were completed using Python (v3.12.12; Python Software Foundation, Wilmington, DE, USA) in the Google Colab environment (Google LLC, Mountain View, CA, USA). Data cleaning and restructuring were done using the Python package Pandas (v2.2.2). Figures were generated using Plotly (v5.24.1; Plotly Technologies Inc., Montréal, Quebec, Canada), and statistics were run using Statsmodels (v0.14.6; Statsmodels Development Team, University of Washington, Seattle, WA, USA). Figures were edited and rendered using Adobe Illustrator (v29.5.1; Adobe Inc., San Jose, CA, USA).

## Results

Overall procedure volume trends

**Figure 1:**
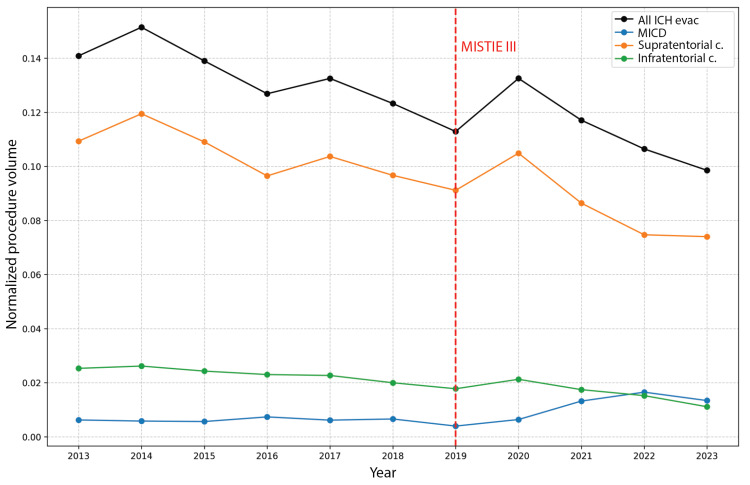
Normalized ICH evacuation volumes before and after the publication of MISTIE III The year is on the x-axis, and the normalized procedure volume, i.e., the percentage of all neurosurgical procedures that are ICH evacuation procedures, is on the y-axis. The normalized procedure volumes were calculated by dividing the number of ICH evacuation procedures by the total number of primary CPT codes billed for all neurosurgical procedures. ICH: intracerebral hemorrhage, Evac: evacuation, MISTIE III: Minimally Invasive Surgery Plus Alteplase for Intracerebral Hemorrhage Evacuation III, MICD: minimally invasive catheter drainage, CPT: current procedural terminology

The total volume of ICH evacuations ranged from 3,387 to 4,863 procedures per year from 2013 to 2023. The normalized volume of all ICH evacuation procedures decreased overall by 30.06% from 2013 to 2023. During this period, the normalized MICD volumes increased by 114.96%, while the normalized volumes of supratentorial and infratentorial craniotomies for ICH evacuation decreased by 32.30% and 56.08%, respectively. The normalized volumes for all ICH evacuation procedures are in Figure 1 and Table 1.

**Table 1:** ICH evacuation procedure volumes over time The ICH-normalized procedure volumes were calculated by determining the percentage of all neurosurgical procedures (primary CPT codes) that are ICH evacuations. This normalizes overall volume trends that are independent of ICH evacuation specifically. * Publication year of MISTIE III ICH: intracerebral hemorrhage, MISTIE III: Minimally Invasive Surgery Plus Alteplase for Intracerebral Hemorrhage Evacuation III, MIS: minimally invasive catheter drainage, CPT: current procedural terminology

Year	Total number of all neurosurgical procedures	All ICH evacuation		MICD		Supratentorial craniotomy		Infratentorial craniotomy	
		Number of procedures	Normalized volume (percentage of all services (%))	Number of procedures	Normalized volume	Number of procedures	Normalized volume	Number of procedures	Normalized volume
2013	3,368,322	4,746	0.141	210	0.00623	3,683	0.109	853	0.0253
2014	3,210,568	4,863	0.151	187	0.00582	3,836	0.119	840	0.0262
2015	3,328,184	4,627	0.139	188	0.00565	3,630	0.109	809	0.0243
2016	3,503,917	4,446	0.127	258	0.00736	3,381	0.0965	807	0.0230
2017	3,601,039	4,773	0.133	222	0.00616	3,734	0.104	817	0.0227
2018	3,702,366	4,564	0.123	244	0.00659	3,580	0.0967	740	0.0200
2019*	3,797,484	4,288	0.113	151	0.00398	3,462	0.0912	675	0.0178
2020	3,191,651	4,231	0.133	204	0.00639	3,348	0.105	679	0.0213
2021	3,313,122	3,879	0.117	438	0.01322	2,863	0.0864	578	0.0174
2022	3,269,642	3,481	0.106	540	0.01652	2,443	0.0747	498	0.0152
2023	3,335,438	3,287	0.0985	447	0.01340	2,469	0.0741	371	0.0111

Procedure volume trends in response to the MISTIE III trial

Following the publication of the MISTIE III trial results in 2019, we observed significant changes in all ICH evacuation procedure types among the Medicare population. From 2013 to 2019, normalized MICD volumes increased slightly overall, although with some fluctuations (β_pre_ = 0.0001, p = 0.022). In the year following 2019, the normalized procedure volume was lower than expected based on the prior trend (β_post1_ = -0.0185, p < 0.001). However, in the long term, the overall trend was much higher after 2019 compared to before 2019 (β_post2_ = 0.0028, p < 0.001) (Tables 1-2).

**Table 2:** ITS analysis of the impact of RCTs on normalized ICH evacuation procedure volumes β values reported as β (95% CI). * p < 0.05, *** p < 0.0001, no asterisk is for non-significant (p ≥ 0.05) ICH: intracerebral hemorrhage, MISTIE III: Minimally Invasive Surgery Plus Alteplase for Intracerebral Hemorrhage Evacuation III, MIS: minimally invasive catheter drainage, CI: confidence interval, ITS: interrupted time series

ICH evacuation procedure	Pre-RCT		Post-RCT (immediate)		Post-RCT (long-term)	
	β_pre_	p	β_post1_	p	β_post2_	p
All	-0.0045 (-0.006 to -0.003)	<0.001***	0.0105 (-0.029 to 0.050)	0.60	-0.0010 (-0.006 to 0.004)	0.68
							MICD	0.0001 (0.0 to 0.0001)	0.022*	-0.0185 (-0.027 to -0.010)	<0.001***	0.0028 (0.002 to 0.004)	<0.001***
Supratentorial craniotomy	-0.0035 (-0.005 to -0.002)	<0.001***	0.023 (-0.004 to 0.051)	0.10	-0.0029 (-0.006 to 0.0)	0.091
Infratentorial craniotomy	-0.0011 (-0.001 to -0.001)	<0.001***	0.0057 (-0.004 to 0.015)	0.23	-0.00080 (-0.002 to 0.0)	0.18

The normalized volumes for supratentorial and infratentorial craniotomies for ICH evacuation showed a decreasing trend from 2013 to 2019 (β_pre_ = -0.0035, p < 0.001; β_pre_ = -0.0011, p < 0.001, respectively). Immediately following 2019, these volumes were higher than expected based on the prior trend, albeit nonsignificantly (β_post1_ = 0.023, p = 0.10; β_post1_ = 0.0057, p = 0.23, respectively). However, in the long term, the share of supratentorial and infratentorial craniotomies declined, albeit nonsignificantly (β_post2_ = -0.0029, p = 0.091; β_post2_ = -0.00080, p = 0.18, respectively) (Tables 1-2).

Lastly, the normalized volumes of all ICH evacuation procedures, including MICD and open craniotomies, declined from 2013 to 2019 (β_pre_ = -0.0045, p < 0.001). In the year following 2019, these volumes were higher than expected based on the prior trend (β_post1_ = 0.0105, p = 0.60). In the long term, the trend continued to decline, yet nonsignificantly (β_post2_ = -0.0010, p = 0.68) (Tables 1-2).

The overall trends identified in the normalized volume results were the same as those in the raw volume results. Thus, using normalized volume trends did not result in different results.

Summary

Our results show that, following the publication year of MISTIE III, the normalized MICD volumes increased from 2019 to 2023, exceeding the level expected by the pre-2019 trend. In contrast, the normalized volumes of both supratentorial and infratentorial craniotomies for ICH evacuation decreased following 2019.

## Discussion

Within our study period, MISTIE III was the only randomized trial evaluating MIS for ICH evacuation. Out of the 499 patients included in the primary statistical analysis, 250 were in the MIS plus thrombolysis group, while 249 were managed according to standard medical protocol. The MISTIE procedure relies on a burr hole placement, stereotactic placement of a rigid cannula into the hematoma, and drainage. Then, a catheter was placed and used to deliver alteplase every eight hours postoperatively until a residual hematoma volume of ≤15 mL was achieved. In our analysis, we analyzed volumes of the MISTIE III procedure over time with CPT code 61156 and refer to this as MICD. Modified intention-to-treat analysis revealed that 45% of patients in the MICD group and 41% in the standard medical group achieved an mRS score of 0-3 one year after stroke diagnosis. However, this difference was not statistically significant (p = 0.33). It is important to note that while the results of MISTIE III did not show improvements in the primary outcome of functional independence, the investigators found that secondary outcomes, including all-cause mortality (p = 0.037) and risk difference with increased clot size (p = 0.03), favored MICD. Increased intracranial pressure and low cerebral perfusion pressure events were also less frequent in the MICD group (p = 0.01 and p = 0.04, respectively).

Furthermore, the authors concluded that MICD for ICH evacuation was safe [8]. Our analysis suggests a significant increase in MICD procedures for ICH evacuation in the four years following the publication year of the MISTIE III results. This trend was higher than expected relative to the pre-2019 trend. Although MICD was not associated with improved primary functional outcomes, the reported secondary improvements may help explain the observed trend.

We searched the neurosurgical literature to help provide context on trends in ICH evacuation volumes before 2013. Simon et al. investigated volumes of supratentorial (61313) and infratentorial (61315) ICH evacuation following the results of the 2006 STICH trial [12]. The STICH trial compared early craniotomy with medical management for ICH and found no significant benefit of surgery for the primary outcome [5]. Simon et al. used Medicare data from 2002 to 2011. They found that craniotomy volumes for ICH evacuation decreased consistently during this period, but the period after publication of the STICH trial did not show an acceleration in this decline. This study also provides 10 years of Medicare data that is no longer available, which allowed us to incorporate it into our current analysis to contextualize trends over a longer period. They reported that supratentorial craniotomy evacuation volumes declined from approximately 2,200 in 2002 to 1,750 in 2011. Infratentorial craniotomy evacuation volumes remained steady around 500-600 procedures per year during this time period [12]. Our analysis suggests that this decline in supratentorial craniotomy evacuation volumes extended into 2023. Our analysis of Medicare data after 2013 showed a greater decline in normalized infratentorial craniotomy evacuation volumes than the data from Simon et al.

During our data period from 2013 to 2022, other factors, including widely disseminated guideline changes, may have influenced the observed changes in ICH evacuation procedure volume outside of MISTIE III. The 2022 AHA/ASA guidelines for the management of spontaneous ICH state that MIS via stereotactic or endoscopic approaches "is safe and may be useful to reduce mortality" and "is considered to improve functional outcomes compared with conventional craniotomy." Importantly, these guidelines note that conventional craniotomy showed "no benefit in functional outcome" across the STICH trial series, which may help explain the continued decline in craniotomy volumes and the corresponding increase in MIS in recent years [9]. Although we observed a decline in both ICH evacuation types (MICD and craniotomy) from 2022 to 2023, we will ultimately need more data to evaluate the long-term impact of these AHA/ASA guidelines.

Murthy et al. published a retrospective analysis of the AHA Get With The Guidelines-Stroke (GWTG-Stroke) registry in 2025. This registry contains data on patients treated with MIS evacuation for nontraumatic ICH from 2011 to 2021. Of the patients receiving surgery, the majority underwent craniotomy (n = 7,191/9,004 (79.86%)), while a small minority underwent MIS evacuation (n = 703/9,004 (7.81%)). This reflects our findings that craniotomy volumes, specifically supratentorial craniotomies, were much higher than those of MIS procedures throughout 2013 to 2023. These authors found that compared to patients with ICH who did not undergo surgery, MIS was associated with lower odds of in-hospital mortality and higher odds of discharge to inpatient rehabilitation [13]. The favorability of MIS versus craniotomy for ICH treatment has also been corroborated by a 2025 review by Woehl et al., whose meta-analysis included 10 RCTs involving more than 2,000 patients. The authors found that MIS was superior to craniotomy in improving functional outcome, limiting intraoperative blood loss, and shortening operative time (p < 0.01). Thus, we posit that the cumulative effect of several other RCTs, in addition to MISTIE III, has driven trends towards MIS and away from craniotomy over the past several years [14].

The Early Minimally Invasive Removal of Intracerebral Hemorrhage (ENRICH) trial, published in 2024, represents one such development. ENRICH compared minimally invasive endoscopic hematoma evacuation using the Myriad evacuation device with medical management alone for supratentorial ICH [15]. Similar to previous studies such as STICH II, which narrowed the study population to identify subgroups that may exhibit positive results [6], ENRICH analyzed only lobar or anterior basal ganglia hemorrhages [15]. Their study found improved utility-weighted functional outcomes in MIS compared with the control group, providing the first concrete evidence of the superiority of minimally invasive surgical intervention in ICH treatment over standard medical management [15]. From 2019 to 2022, ongoing trials such as ENRICH may have increased interest in MICD for treating ICH and contributed to the increase in normalized volumes we observed following MISTIE III.

Additionally, ongoing development and marketing of new devices after MISTIE III could partially explain the post-MISTIE III increase in normalized MICD procedure volumes. The MIND trial from 2025, which also examined the subgroup of supratentorial ICH patients, found no significant benefits of minimally invasive hematoma evacuation via a burr hole compared with medical management alone [16]. All patients in the MIND trial were treated with the Artemis Neuro Evacuation Device [16], whereas the ENRICH trial used the Myriad evacuation device [15]. The development and evaluation of multiple devices in ongoing trials may have increased interest in MICD for treating ICH from 2019 to 2022.

One of the strengths of our analyses is that we used the most recently released Medicare dataset. This allowed us to build on prior studies that analyzed ICH evacuation volumes, such as Simon et al., which included data up to 2011 and did not include any data on MIS evacuation, a newer technique [12]. To our knowledge, our study is the first to specifically analyze how ICH evacuation procedure volumes have changed after the publication of the MISTIE III trial, with a specific focus on MICD. An additional strength of our study is that we used normalized procedure volumes, which statistically correct for changes affecting the entire surgical landscape, such as the COVID-19 pandemic [17].

A notable limitation of our analysis is our focus on the publication of the MISTIE III trial results as the primary driver of change in ICH surgical treatment. Therefore, it is not possible to prove that the results of MISTIE III directly caused the trends we observed in the dataset. Even though we used normalized volumes, other factors during those publication years could have influenced the procedure volumes, such as non-RCT publications, additional guidelines published during this time period (as discussed above), or technological advancements. Additionally, using normalized volumes does not account for policies implemented during the COVID-19 pandemic that may have differentially affected certain neurosurgical procedures compared with others. Moreover, the ITS model uses six pre-trial data points (2013 to 2018) and four post-trial data points (2020-2023). This is a limited number of data points, which may limit the conclusions that can be drawn from the analysis. The model also assumes that there are no major concurrent interventions during the timeline, which we found to be untrue due to events such as the publication of the 2022 AHA/ASA guidelines, as discussed [9]. While it may be informative to conduct another ITS analysis with 2022 as the data knot, there is insufficient post-2022 Medicare data available to do so. This opens the opportunity for future analyses once that data is made available.

Additionally, our analysis is limited to the individual CPT codes that we included. Although we identified 61156 as the closest representation of the MISTIE III MICD procedure, some institutions may bill 61313 for the MISTIE III procedure. Therefore, the trends we identified should be interpreted in the context of their respective CPT codes rather than as direct comparisons between MICD and craniotomy.

Additional limitations concern the use of our Medicare dataset. The dataset does not contain patient outcomes and is limited to adults in the US with Medicare coverage. Many of the patients enrolled in the MISTIE III trial were at least 65 years old and thus qualified for Medicare, but the mean age for both the MICD and medical management groups was 62 [8]. Performing these analyses on a more comprehensive dataset is a potential avenue for future research.

## Conclusions

Following the publication of the MISTIE III results in 2019, which showed no benefit of MICD, normalized MICD procedure volumes increased significantly relative to the pre-2019 trend. In contrast, the normalized volume of craniotomy ICH evacuation procedures did not change significantly. The contrast between the MISTIE III results and the normalized procedure volumes could be due to the positive secondary outcomes of the MISTIE III trial, preliminary results from ongoing trials, and the development and marketing of new devices that may have increased neurosurgeons' interest in MICD. However, changes in procedure volume rates should be interpreted with caution, as they are correlated with the publication date of MISTIE III and are by no means causative. Additionally, several other factors during this period could have affected procedure volumes, including changes to clinical guidelines and technological developments. Once additional Medicare data becomes available, there will be sufficient data to evaluate the potential impact of these factors, providing opportunities for future research.
